# Unveiling the dark side of glucose-regulated protein 78 (GRP78) in cancers and other human pathology: a systematic review

**DOI:** 10.1186/s10020-023-00706-6

**Published:** 2023-08-21

**Authors:** Amos Olalekan Akinyemi, Kendall Elizabeth Simpson, Sunday Faith Oyelere, Maria Nur, Chrispus Mutuku Ngule, Bolaji Charles Dayo Owoyemi, Vivian Adiila Ayarick, Felix Femi Oyelami, Oluwafunminiyi Obaleye, Dave-Preston Esoe, Xiaoqi Liu, Zhiguo Li

**Affiliations:** 1 Department of Toxicology and Cancer Biology, University of Kentucky, Lexington, USA; 2 Department of Biology, Heinrich-Heine-Universität, Düsseldorf, Germany; 3 Department of Pharmaceutical Science, University of São Paulo (USP), Sao Paulo, Brazil; 4 Markey Cancer Center, College of Medicine, University of Kentucky, Lexington, USA

**Keywords:** Cancer, GRP78, BiP, Disease, ER stress, ERAD, URP, Chaperon

## Abstract

Glucose-Regulated Protein 78 (GRP78) is a chaperone protein that is predominantly expressed in the lumen of the endoplasmic reticulum. GRP78 plays a crucial role in protein folding by assisting in the assembly of misfolded proteins. Under cellular stress conditions, GRP78 can translocate to the cell surface (csGRP78) were it interacts with different ligands to initiate various intracellular pathways. The expression of csGRP78 has been associated with tumor initiation and progression of multiple cancer types. This review provides a comprehensive analysis of the existing evidence on the roles of GRP78 in various types of cancer and other human pathology. Additionally, the review discusses the current understanding of the mechanisms underlying GRP78's involvement in tumorigenesis and cancer advancement. Furthermore, we highlight recent innovative approaches employed in downregulating GRP78 expression in cancers as a potential therapeutic target.

## Introduction

Glucose-Regulated Protein 78 (GRP78) is a heat shock protein chaperone that has garnered extensive attention in various disease progression (Gopal and Pizzo 2021). GRP78 primarily resides in the lumen of the endoplasmic reticulum, a cellular organelle responsible for protein synthesis and folding (Lee 2001). It plays a vital role in maintaining protein homeostasis by assisting in the assembly and proper folding of newly synthesized proteins (Pyrko et al. 2007).Under normal physiological conditions, GRP78 ensures the efficient folding of proteins and prevents the accumulation of misfolded or unfolded proteins in the endoplasmic reticulum (Fu et al. 2008). However, in response to cellular stress, such as nutrient deprivation, hypoxia, or exposure to toxins, the demand for proper protein folding increases. In these conditions, GRP78 expression is upregulated to meet the heightened protein-folding requirements (Koumenis et al. 2002). Interestingly, in addition to its role within the endoplasmic reticulum, GRP78 has been found to undergo translocation to the cell surface. This cell surface localization gives rise to a distinct form of GRP78 known as cell surface GRP78 (csGRP78) (Misra et al. 2005). The translocation of GRP78 to the cell surface is believed to be a stress response mechanism that allows cells to adapt and survive adverse conditions (Lee 2001).

csGRP78 has been implicated in the development and progression of various types of cancer (Dong et al. 2005). It has been observed that csGRP78 expression is often upregulated in cancer cells compared to normal cells (Dong et al. 2011). This overexpression is associated with several hallmarks of cancer, including increased cell survival, enhanced proliferation, angiogenesis (formation of new blood vessels to support tumor growth), and resistance to chemotherapy and radiation therapy (Sedighzadeh et al. 2021). At the cell surface, csGRP78 interacts with specific ligands, such as hormones, growth factors, and extracellular matrix proteins. These interactions trigger signaling pathways that promote cell survival, activate pro-survival and pro-growth signaling cascades, and contribute to the acquisition of malignant traits by cancer cells (Dong et al. 2005; Fu et al. 2008). Understanding the intricate involvement of GRP78, particularly csGRP78, in cancer biology would therefore be crucial in developing effective therapeutic strategies. Targeting GRP78 or its interactions with ligands holds promise for disrupting the survival and growth pathways that drive cancer progression (Fu et al. 2008). Therefore, numerous research efforts have been directed towards unraveling the underlying mechanisms of GRP78 in cancer and exploring innovative approaches to modulate its expression or function (Ninkovic et al. 2020; Qiao et al. 2020).

In this review, we aim to provide a systematic breakdown of the correlation between GRP78 and various types of cancer. By compiling and analyzing relevant research findings, we seek to elucidate the extensive role of GRP78 in cancer pathogenesis and shed light on recent advances in downregulating GRP78 as a potential therapeutic avenue.

### Discovery and types of GRPs

In the 1970s, researchers (Pouysségur et al. 1977; Hightower 1980) identified a group of proteins that were constitutively produced and activated in response to glucose deprivation, and they were given the moniker glucose-regulated proteins (GRPs). One of the most extensively studied GRPs is GRP78. The chaperone protein GRP78 was first identified and described by Stone et al. in 1974 (Stone et al. 1974). Initially, in 1976, it was believed that GRP78 was a 73 kDa membrane protein associated with viral transformation. However, subsequent studies by Shiu et al. in 1977 demonstrated that this membrane protein was primarily involved in glucose regulation within cells rather than viral transformation (Shiu et al. 1977).

Over the years, the understanding of GRPs has expanded, and various types of GRPs have been identified with their distinct functions. These GRPs act as chaperones and are activated in response to cellular stress. Here are some of the key types of GRPs and their important functions (Table 1):

**Table 1:** Types of GRPs with their important functions

GRP type	Structure	Functional description	References
GRP75 (mtHSP70)		Human HSPA9 encodes GRP75, a 75-kDa protein that is otherwise known as Mortalin. GRP75 is localized primarily in the mitochondria and functions as a chaperone for mitochondrial proteins. It could also be found on the plasma membranes, and in the endoplasmic reticulum and mitochondria-associated ER membrane. The different subcellular localization of GRP75 confers on it different physiological functions which include regulation of cell cycle, response to stress, generation of metabolic energy, membrane transportation, regulation of the folding, assembly, and maintenance of mitochondrial proteins. In many malignancies, GRP75 expression is increased, and several effects, such as inactivating the tumor suppressor p53, regulating apoptosis, and enhancing cancer stemness. Its other pathophysiological functions include stimulation of the proliferation and immortality of cancer cells and facilitation of cancer resistance to chemotherapy	(Wadhwa et al. 2002, 1993; Li et al. 2022; Yoon et al. 2022)
GRP78 (BiP)		GRP78, also known as immunoglobulin heavy chain-binding protein (BiP), is a chaperone protein that is primarily located in the endoplasmic reticulum (ER) of eukaryotic cells. It plays a crucial role in protein folding and quality control, ensuring proper protein maturation and preventing the accumulation of misfolded or unfolded proteins. GRP78 is a member of the heat shock protein 70 (HSP70) family and is highly conserved across species. Protein folding in the ER is a complex process, and GRP78 acts as a master regulator by binding to unfolded or misfolded proteins, preventing their aggregation, and promoting their correct folding. It possesses an ATPase activity, which allows it to cycle between its ATP-bound state (active) and its ADP-bound state (inactive), facilitating the binding and release of client proteins. In addition to its role in protein folding, GRP78 is involved in maintaining ER homeostasis. It interacts with transmembrane sensor proteins such as PERK (protein kinase RNA-like ER kinase), ATF6 (activating transcription factor 6), and IRE1 (inositol-requiring enzyme 1), which are key components of the unfolded protein response (UPR). The UPR is an adaptive signaling pathway that is activated in response to ER stress, aiming to restore ER function and alleviate the accumulation of unfolded proteins. GRP78 plays a critical role in activating and regulating the UPR, ensuring that the cellular response to ER stress is appropriately controlled	(Shiu et al. 1977; Ni and Lee 2007; Li et al. 2008; Hendershot et al. 1995; Pfaffenbach and Lee 2011; Zhu and Lee 2015; Wang et al. 2009; Gardner et al. 2013; Walter and Ron 2011)
GRP94		GRP94, otherwise known as tumor rejection antigen 1TRA1, is predominantly located in the endoplasmic reticulum and functions as a chaperone for secretory and membrane proteins. It is a cellular protein that is rapidly synthesized during glucose depletion, oxidative stress, distorted ER actions, accumulation of misfolded proteins, and ER calcium depletion. It is divided into four major domains: The N terminal domain, the middle domain, the charged linker region and the C-terminal domain. GRP94 has three main functions, first, as a molecular chaperon essential for proper folding and quality control of client proteins. It is also a calcium regulator in the ER where it supports calcium ion storage and stabilize the intracellular concentration of calcium ion. Lastly, GRP94 appears to be a key regulator of immune system. Just like other GRPs, GRP94 upregulation is documented in different cancers. GRP94 plays significant role in cancer progression and metastasis by promoting cancer cell proliferation, tumor growth, tumor angiogenesis and cancer cell invasion and metastasis. Some reports also show that GRP94 facilitates tumor resistance to therapy	(Koch et al. 1986; Mazzarella and Green 1987; Maki et al. 1990; Johnson 2012; Ni and Lee 2007; Schild and Rammensee 2000; Kim et al. 2021; Duan and Xin 2020)
GRP170		GRP170 is the largest member of GRPs. Its synthesis is induced by glucose deprivation and other stress inducers such as hypoxia, calcium imbalance, ischemia, inhibition of proteasome and non-steroidal anti-inflammatory drugs. GRP170 is structurally related to GRP78 as they both have N-terminal nucleotide binding domain, substrate binding domain, and an alpha-helical C terminus domain. GRP170 facilitates protein folding, assembly, and transport across membranes. It also plays a role in antigen presentation and immune responses. More so, it is proven to be a highly efficient ATP-binding protein in the microsome. Its role as a nucleotide exchange factor is recently reported. During the development and progression of cancers, GRP170 plays crucial roles. For instance, it contributes to tumor invasiveness, tumor survival, angiogenesis, and tumor metastasis	(Chen et al. 1996; Kuwabara et al. 1996; Sciandra et al. 1984; Wang et al. 2014)
GRP58 (ERp57)		Glucose regulated protein 58 kDa is an endoplasmic reticulum protein that is secreted in response to stress such as glucose shortage in living system. GRP58 is structurally related to disulfide isomerase. Although it localizes majorly in the ER, it is also found in the cytosol and nucleus. Within the cytosolic portion, GRP58 functions as a chaperone during the process of transduction and activation of transcriptional signaling. It facilitates the formation of complexes between STAT3 and scaffolding proteins. Once inside the nucleus, STAT3 and these proteins are recognized as integral components of matrix proteins, which play a vital role in attaching DNA to the nuclear matrix and creating DNA loops. Under normal circumstances, GRP58 binds with calreticulin and calnexin to form complexes that co-interact with glycoproteins in the endoplasmic reticulum, thereby acting as chaperone in the anabolism of glycoproteins. However, both upregulation and downregulation of GRP58 are involved in carcinogenesis. Upregulation of GRP58 is widely reported in various cancers such as ovary, stomach, lung, uterus, and breast cancers. Interestingly, its downregulation is also identified in esophageal, renal cell, gastric and cervical cancers	(Hafiza and Latifah 2014; Celli and Jaiswal 2003; Hirano et al. 1995; Frickel et al. 2004)

A comprehensive literature search using the terms "GRP78" or "BiP" reveals a vast number of publications on the topic, reflecting the extensive research interest in GRP78. Most references cited in this article are derived from reliable sources such as PubMed, Web of Science, and reputable scientific publishers like Elsevier and Wiley. This study provides a deeper insight into the role of GRP78 and various diseases, particularly cancer. GRP78 has been implicated in the pathogenesis and progression of various cancers, highlighting its significance as a potential therapeutic target (Luo et al. 2018). Investigating the subcellular processes and functions of GRP78 offers valuable insights into the development of novel strategies for the treatment of associated diseases.

### Description of GRP78 coupled with its roles

**Fig. 1:**
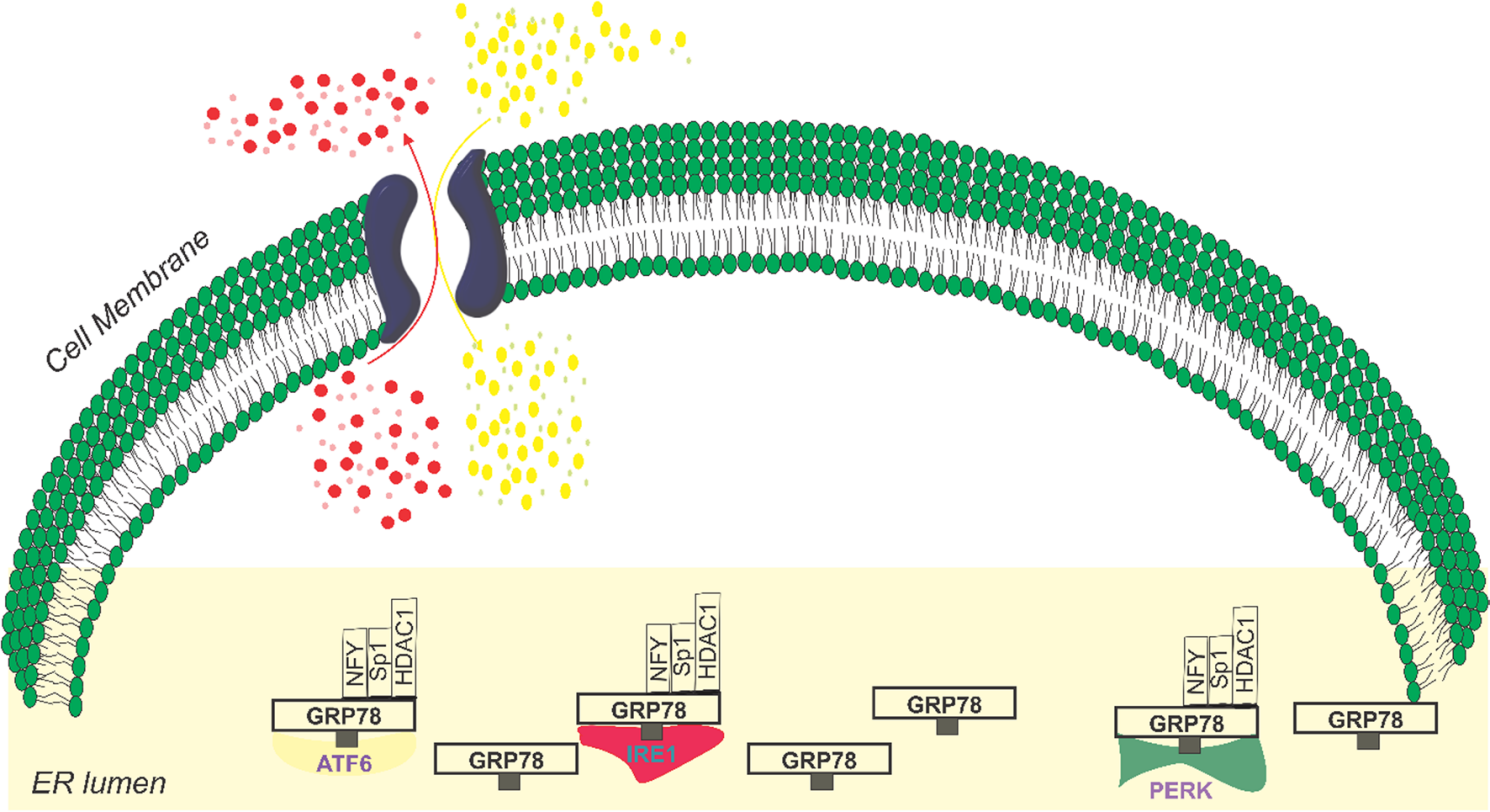
Illustration of the inactive state of GRP78 and the presence of stress response elements (ERES) positioned upstream of the TATA element. In cells that are not experiencing stress, the ERES region is bound by NFY, SP1, and HDAC1, leading to the maintenance of low transcription levels for GRP78. Additionally, GRP78, located within the lumen of the endoplasmic reticulum (ER), interacts with the ER stress sensors (IRE1, ATF6, and PERK) and acts as a signaling component for the unfolded protein response (UPR)

**Fig. 2:**
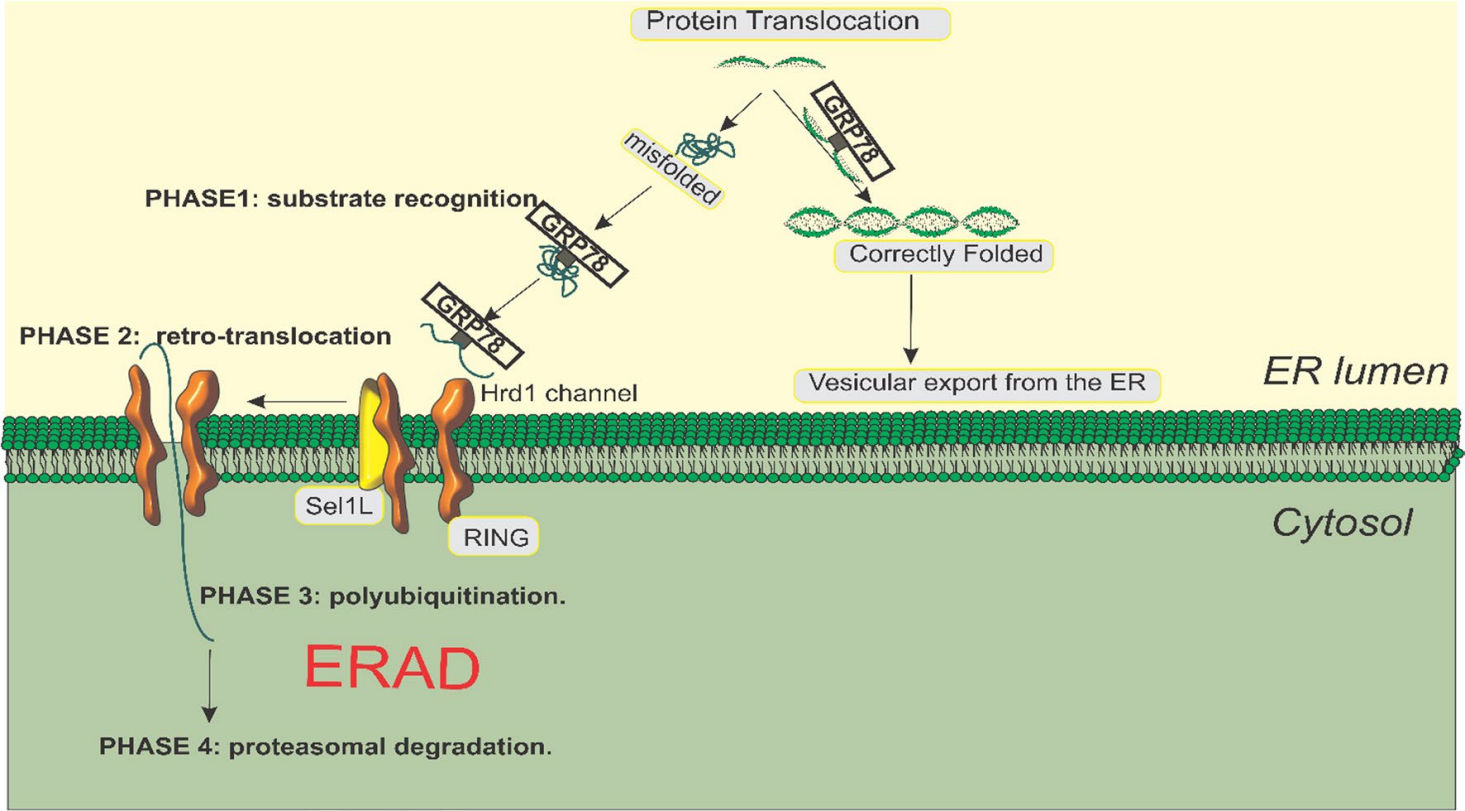
Representation of the general mechanism by which GRP78 functions within the endoplasmic reticulum (ER). GRP78 plays a crucial role in assisting ER proteins by promoting their stability and facilitating the process of protein folding. This is achieved through its interaction with misfolded proteins and unassembled complexes, thereby initiating the ER-associated degradation (ERAD) pathway. Within this pathway, GRP78 targets the misfolded proteins for degradation through a series of events that involve ubiquitination and subsequent proteasomal degradation

GRP78, also known as glucose-regulated protein 78, is a chaperone protein that primarily resides in the lumen of the endoplasmic reticulum (ER) due to its carboxyl KDEL retention motif, but it can also be found on the cell surface (Lee 2014). It is encoded by the Hsp5a gene and belongs to the heat shock protein-70 (Hsp70) family. Although GRP78 is the most common member of the Hsp70 family, it lacks a heat shock element in its promoter, preventing activation by heat shock (Casas 2017). In normal cell states, GRP78 remains inactive while bound to transmembrane stress detectors of the unfolded protein response (UPR) pathway, including PRK-like endoplasmic reticulum kinase (PERK), inositol-requiring enzyme (IRE1), and activating transcription factor 6 (ATF6) (Pfaffenbach and Lee 2011) (Fig. 1). This binding keeps GRP78 in an inactive state, ready to respond to ER stress. The regulation of GRP78 involves the binding of nuclear transcription factor Y (NFY), SP1, and histone deacetylase 1 (HDAC1) to stress response elements (EREs) present in its promoter region, thereby maintaining its low basal transcription level (Fig. 1) (Yoshida et al. 1998; Roy and Lee 1999; Baumeister et al. 2005). Interestingly, GRP78 plays a dual role within the endoplasmic reticulum. Firstly, it functions as a resident chaperone, facilitating proper protein folding and preventing protein aggregation. This chaperone activity of GRP78 ensures the correct folding and assembly of newly synthesized polypeptides. Secondly, GRP78 acts as a key regulator of the UPR pathway. During ER stress, the accumulation of unfolded proteins sequesters GRP78 away from PERK, IRE1, and ATF6, thereby activating these transmembrane proteins (Fig. 2) (Hendershot 2004). The UPR is a cellular response mechanism that aims to restore ER homeostasis under stress conditions. It is divided into two phases: the early, pro-survival UPR, which activates adaptive responses to mitigate ER stress, and the late, pro-apoptotic UPR, which triggers cell death if ER stress becomes overwhelming(Roller and Maddalo 2013a). It is noteworthy that the persistent activation of the UPR pathway and the dysregulation of GRP78 have been implicated in various malignancies. Genes upstream or downstream of the UPR are frequently upregulated in cancer cells, suggesting that the sustained activation of this pathway provides tumors with a growth advantage(Roller and Maddalo 2013a).

**Fig. 3:**
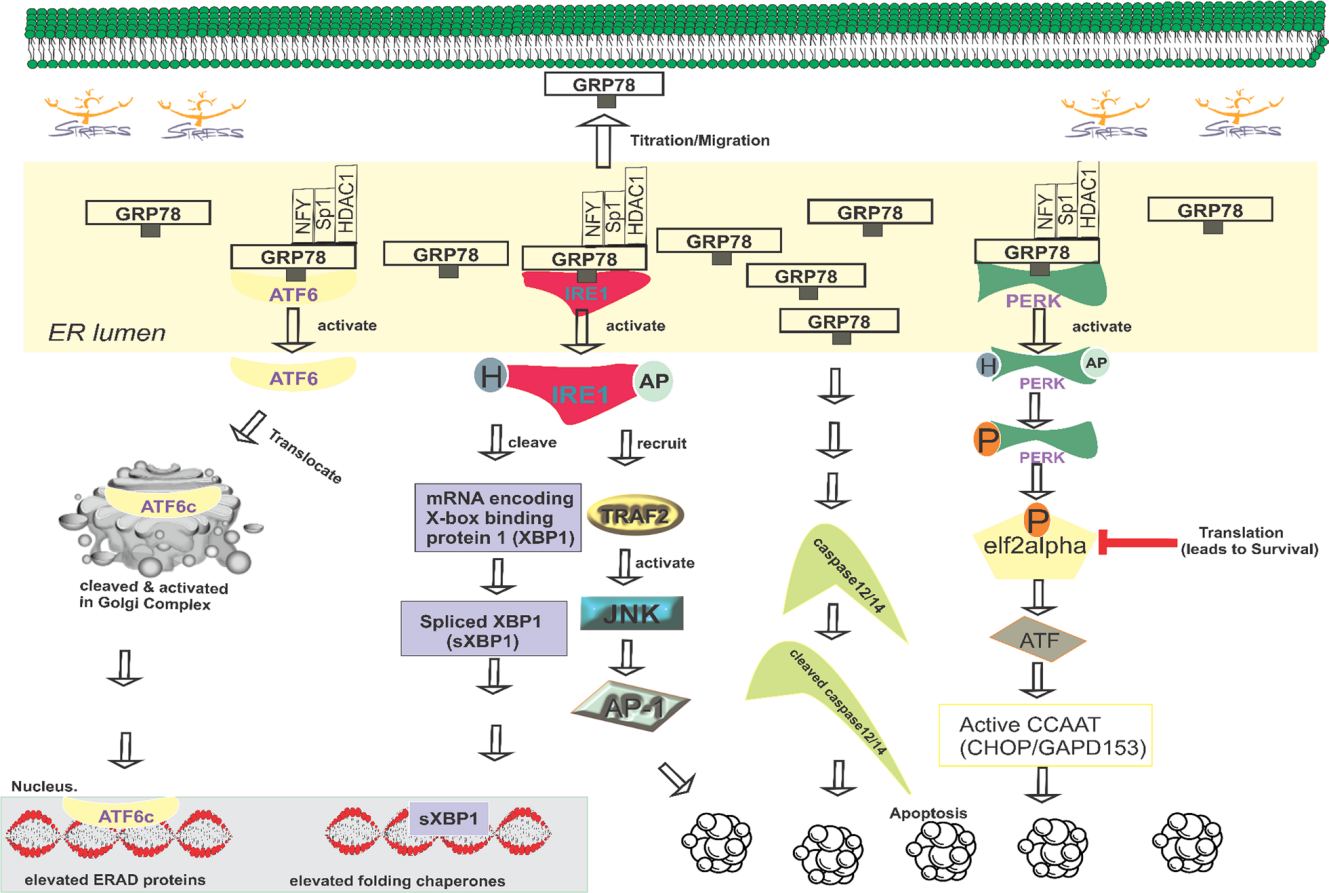
A proposed comprehensive pathway illustrating the involvement of GRP78 as a central regulator in the cellular response to endoplasmic reticulum (ER) stress, known as the unfolded protein response (UPR). In this pathway, GRP78 acts as a master regulator, coordinating the UPR signaling cascade

Studies investigating the transcriptional activation mechanism of GRP78 have contributed to the identification of novel intracellular signaling pathways that transmit ER stress signals to the nucleus, initiating the transcription of genes involved in the unfolded protein response (UPR) (Luo and Lee 2013; Mori 2000; Chang et al. 1989). Figure 3 illustrates the cascade of downstream pathways initiated by UPR stress sensors when unfolded peptides accumulate. Upon activation, PERK forms homodimers and undergoes autophosphorylation, enabling the alpha subunit of eukaryotic translation initiation factor 2 (elf2) to be phosphorylated (Ibrahim et al. 2019; Li et al. 2008; Liu et al. 2020). Phosphorylated elf2 inhibits translation initiation, triggering the activation of the transcription factor ATF4 and its target gene C/EBP homologous protein (CHOP). CHOP, in turn, induces apoptosis by downregulating BCL-2 (an antiapoptotic outer mitochondrial membrane protein) and activating the expression of Bax and Bim genes (Roller and Maddalo 2013a). IRE1 also homodimerizes and auto phosphorylates before cleaving and splicing the mRNA encoding X-box binding protein 1. Additionally, IRE1 recruit’s tumor necrosis factor α receptor-associated factor 2 (TRAF2), which activates JUN amino-terminal kinases (JNK), ultimately leading to apoptosis. Furthermore, ATF6 translocate to the Golgi apparatus after dissociation from GRP78 and subsequently undergoes cleavage, allowing its active form, cATF6, to enter the nucleus. In the nucleus, cATF6 acts as a transcription factor, upregulating proteins essential for enhancing the ER's capacity for protein folding (Ibrahim et al. 2019). The pathways mediated by PERK, IRE1, and ATF6 intersect with each other and elicit cellular responses that can be both pro-survival and pro-apoptotic. Importantly, the induction of GRP78 transcription is not solely triggered by ER stress. Some scholarly articles have demonstrated the activation of GRP78 transcription by histone deacetylase inhibitors (HDAC1) without concurrently inducing the overall stress response (Baumeister et al. 2009). Additionally, impaired autophagy in tumor cells has been shown to upregulate ER chaperones in response to metabolic stress (Mathew et al. 2009).

**Fig. 4:**
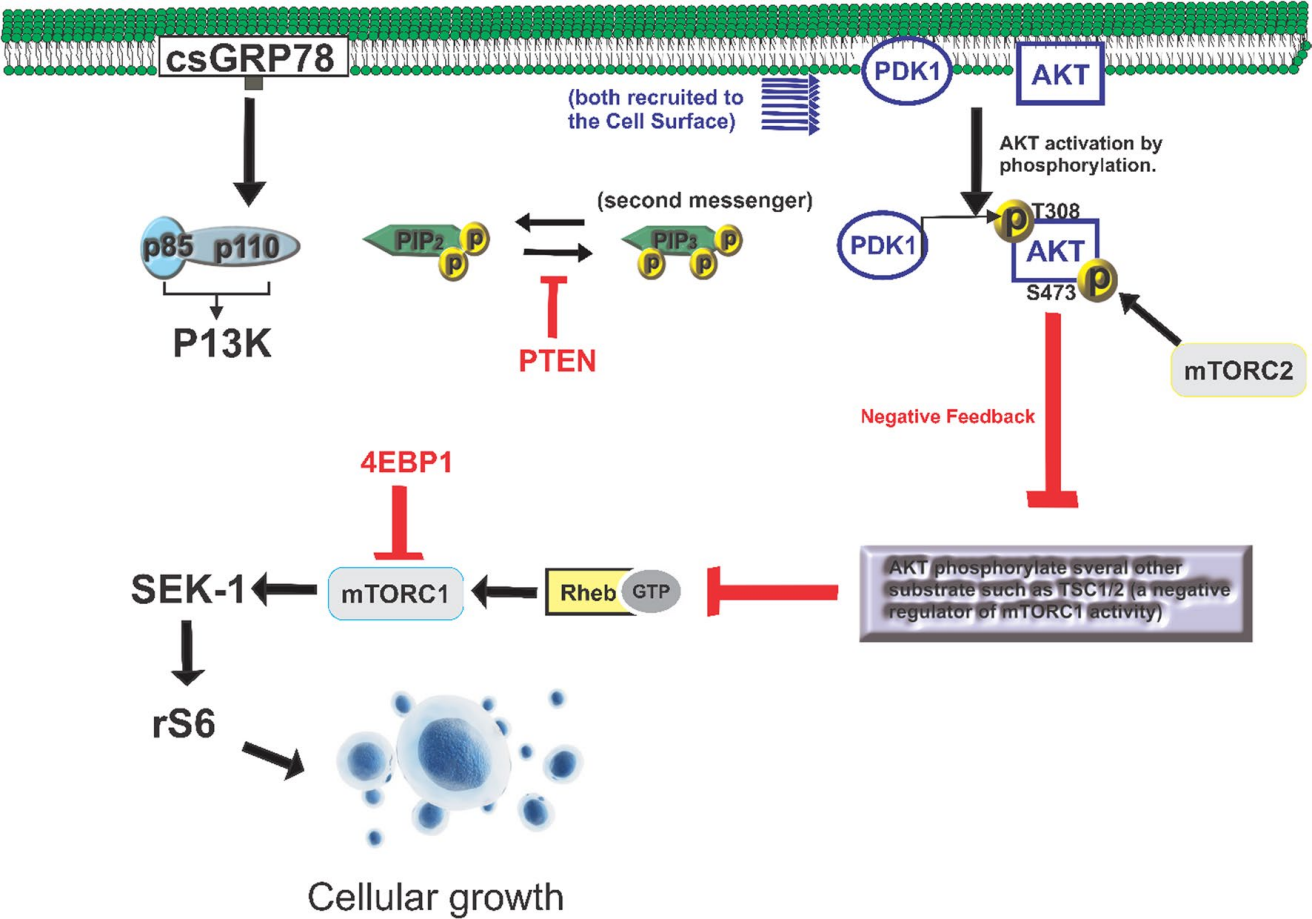
A breakdown of cell surface GRP78 (csGRP78) roles in AKT/P13K/ mTOR pathway. AKT and phosphoinositide-dependent kinase-1 (PDK1) are both drawn to the membrane after PI3K produces the second messenger PIP3. Due to proximity, PDK1 can phosphorylate AKT at position T308 of the activation loop (T-loop). The rapamycin-resistant mTOR complex 2 then phosphorylates AKT at residue S473 of the hydrophobic motif (mTORC2), a key step in the complete activation of AKT's kinase activity. TSC2, a negative regulator of mTORC1 activity, is one of several additional substrates that AKT itself has the ability to phosphorylate

In tumor microenvironments where GRP78 is overexpressed, it localizes to the cell membrane surface (Roller and Maddalo 2013b). Referred to as cell surface GRP78 (csGRP78), this protein plays a significant role in various signaling events and acts as a co-receptor in studies involving different tumor types, influencing tumor cell survival, proliferation, and motility. The relocation of GRP78 to the cell surface has been strongly associated with drug resistance and cellular transformation (Roller and Maddalo 2013b; Chen et al. 2022). Additionally, alterations in cell surface GRP78 have been shown to impact the behavior of cancer stem cell populations in diverse tumor types (Chen et al. 2018a, b). One well-studied pathway involving cell surface GRP78 is its interaction with the tumorigenic PI3K/AKT pathway, facilitated by complex formation with PI3K (phosphoinositide-3-kinase) (Lee et al. 2022a) (Fig. 4). This interaction has been observed in prostate cancer (Zhang et al. 2013). In a study conducted by Zhang et al. in 2013 (Zhang et al. 2013), they discovered an insertion mutation in the N-terminus domain of GRP78. This mutation resulted in a decrease in complex formation, while the protein's expression and ability to move to the cell surface remained stable, similar to the normal, unmutated protein. The researchers concluded that blocking csGRP78 could present a distinct strategy to inhibit PI3K activity and potentially overcome treatment resistance in cancer cells.

### GRP78 role in various diseases

GRP78, a master chaperone protein of the unfolded protein response (UPR), plays a crucial role in various diseases, mainly cancer. In cancer, GRP78 has been implicated in chemotherapy resistance and the tumor virulence (Cook and Clarke 2015). Tumor cells often face a hostile metabolic environment characterized by low glucose levels, acidity, and nutrient deprivation (Ferraresi et al. 2020; Wang et al. 2009). Interestingly, the UPR, which involves the activation of GRP78, is triggered in response to oxygen or glucose starvation to promote cell survival (Wojtkowiak et al. 2012; Ferraresi et al. 2020; Wang et al. 2009; Koumenis 2006). Elevated expression levels of GRP78 have been observed in initial tumors compared to benign tissues, indicating its involvement in tumor progression and aggressiveness (Wojtkowiak et al. 2012). GRP78 acts as a multifunctional protein in cancer, contributing to diverse cellular processes such as protein folding, ER homeostasis, regulation of apoptosis, and protection against cellular stress (Ferraresi et al. 2020).

**Fig. 5:**
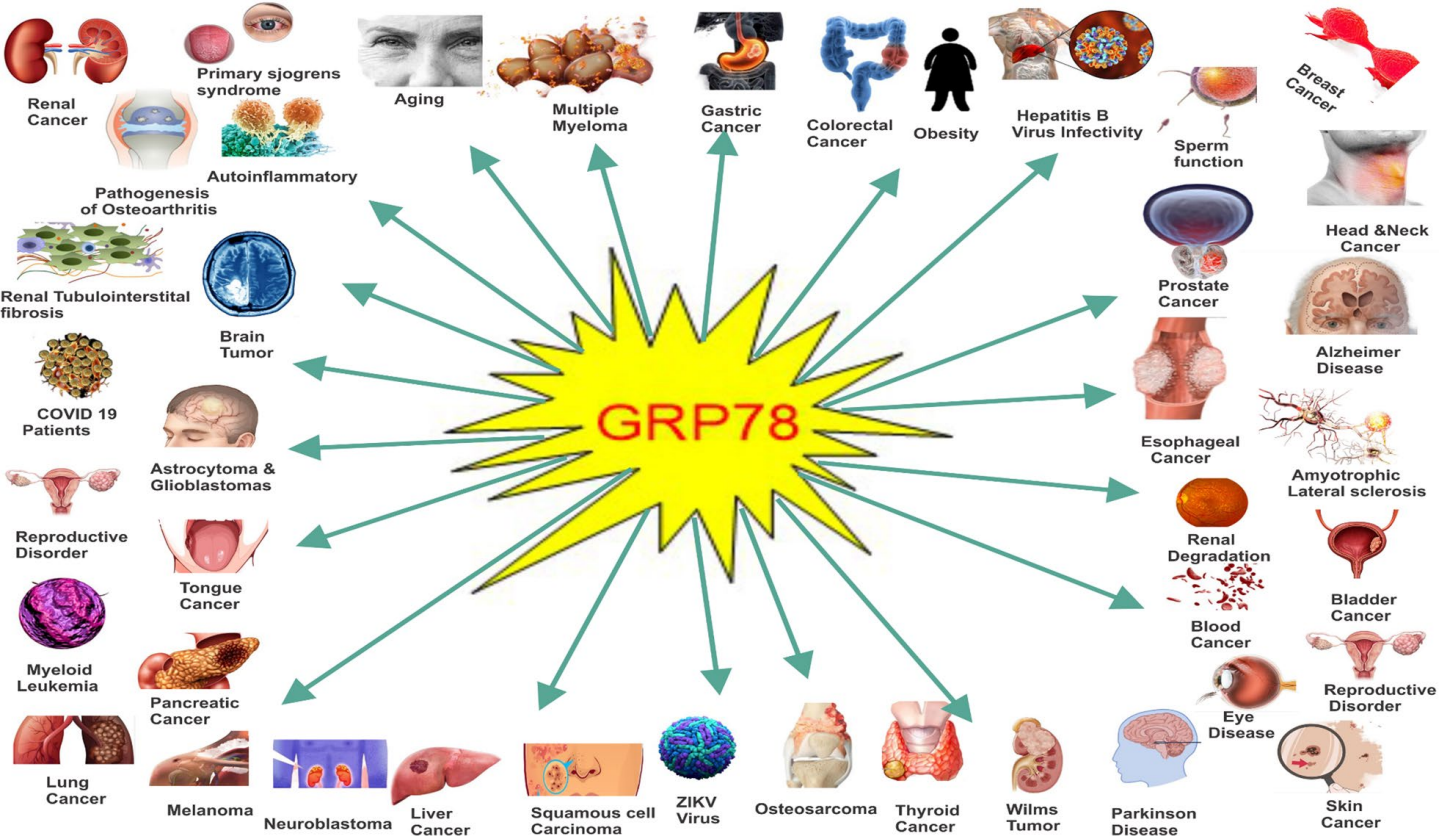
Diagrammatical Illustration showing multifactorial implications of GRP78 in various diseases

The role of GRP78 extends beyond cancer. Here, we highlighted some diseases where GRP78 has been implicated: (Fig. 5 and Table2 below):

**Table 2:** Shows various role of GRP78 in various cancer with mechanisms

	Cancer types/diseases	Function of GRP78	Mechanism of GRP78 involvement	References
1	Multiple Myeloma	Involvement in the regulation of ER stress and the UPR	GRP78 is greatly expressed in multiple myeloma. The expression of MALATI results into polyubiquitinated proteins accumulation, which actuate ERS, induce the expression of GRP78, actuate autophagy and triggers cell apoptosis	(Amodio et al. 2018; Abdel Malek et al. 2015)
2	Gastric Cancer	Cell proliferation, inhibition of apoptosis	GRP78 stimulates cell proliferation, inhibits apoptosis, and confers resistance to chemotherapy. it activates pro-survival pathways, such as the PI3K/AKT pathway, to enhance cell survival and inhibit apoptosis in gastric cancer cells. It also modulates drug efflux pumps and anti-apoptotic pathways, leading to chemoresistance	(Chen et al. 2018a, b; Zhang et al. 2022a; Zheng et al. 2017)
3	Colorectal Cancer	Unfolded protein response, cell survival	GRP78 helps maintain protein homeostasis by activating the UPR in response to endoplasmic reticulum (ER) stress. The UPR regulates protein folding, degradation, and ER-associated degradation. Overexpression of GRP78 in colorectal cancer cells promotes cell survival by modulating the UPR and inhibiting apoptosis. Additionally, GRP78 has been implicated in promoting colorectal cancer metastasis by regulating the epithelial-mesenchymal transition (EMT) process	(Chern et al. 2019; Huang et al. 2021a, b; Xi et al. 2018; Li et al. 2014)
4	Breast Cancer	Cell survival, inhibition of apoptosis	In breast tumor, GRP78 contributes to drug resistance by promoting cancer cells survival. GRP78 activates pro-survival pathways, such as the PI3K/AKT and MAPK/ERK signaling pathways, which promote cell survival and inhibit apoptosis. It also interacts with and stabilizes anti-apoptotic proteins, such as Bcl-2 family members, preventing the activation of apoptotic pathways	(Kuang et al. 2016; Kabakov and Gabai 2021; Dong et al. 2011; Sadeghipour et al. 2023)
5	Head and Neck Cancer	Tumor progression, chemoresistance	The progression, survival and chemo-resistance properties of head and neck cancer are attributed to GRP78. GRP78 promotes the survival of head and neck cancer cells by maintaining lysosomal activities through the help of MUL 1, one of E3 ubiquitin-protein ligases (MUL1-GRP78)	(Kim et al. 2018; Schneider et al. 2022)
6	Prostate Cancer	Androgen receptor signaling, cell survival	GRP78 modulates androgen receptor signaling, promotes cell survival, and contributes to castration resistance in prostate cancer. GRP78 interacts with and stabilizes the androgen receptor, which is crucial for prostate cancer growth and progression. It enhances androgen receptor signaling, leading to increased cell survival and proliferation. Moreover, GRP78 has been implicated in castration-resistant prostate cancer, where it sustains androgen receptor activity even in the absence of androgens, allowing cancer cells to bypass hormone deprivation therapy	(Misra et al. 2004, 2005; Kelber et al. 2009; Luo and Lee 2013)
7	Alzheimer’s Diseases	Protein folding	Alzheimer's disease (AD), a neurodegenerative condition marked by cognitive changes, and memory loss has been linked to GRP78. The hippocampus and temporal cortex of AD patients have twice the amounts of GRP78 compared to normal individual. Evidently, Tau hyperphosphorylation is a clear sign of the tauopathies and Liu shows that overexpression of GRP78 strengthened the interaction between tau and GSK-3 and caused tau hyperphosphorylation via activating glycogen synthase kinase-3 (GSK-3), a key tau kinase in AD brain	(Liu et al. 2016; Casas 2017)
8	Parkinson’s Diseases	Proteostasis, neuroprotection	Lewy bodies and dopaminergic neurons deficiency in the substantia nigra pars compacta (SNc) are two features of Parkinson's disease, an idiopathic movement illness. It has been observed that MPP + treatment induces the translocation of GRP78 from the endoplasmic reticulum (ER) to the nucleus and cytoplasm. This translocation is found to be associated with a significant reduction in the number of cells positive for tyrosine hydroxylase in the SNc	(Ghribi et al. 2003; Bellucci et al. 2011)
9	Aging	Proteostasis, cellular aging	ER chaperones are less responsive to ER stress in old tissues and this is proven by decreased levels and activity of ER chaperones in aging cells; hence, aging renders the quality control mechanism of proteins ineffective. Increased oxidation of numerous important ER chaperones has been linked to this condition, and this is consistent with the mitochondrial free radical theory of aging. In particular, a decline in GRP78 levels has been seen in aging and degenerative disorders	(Nuss et al. 2008; Rabek et al. 2003; Paz Gavilán et al. 2006)
10	Amyotrophic Lateral Sclerosis (ALS)	Proteostasis, ER stress	A progressive neurodegenerative condition called ALS causes the motoneurons in the cerebral cortex, the majority of the brainstem, and the spinal cord to selectively degenerate. Familial ALS is characterized by a variety of mutations, all of which cause protein misfolding and aggregation. Superoxide dismutase 1 (SOD1), TAR DNA-binding protein 43-KDa, FUS, and other genes are affected by these mutations. SOD1 aggregates have been seen in sporadic ALS cases. In microsomal fractions of spinal cords made from mice models of ALS, mutant SOD1 aggregates, produces high molecular weight species, that interacts with GRP78. In addition, SIL1 and Sig1R are a few of GRP78 co-chaperones that are significant in ALS. The fact that SIL1 is mostly expressed in tough motoneurons suggests that it has a role in neuroprotection. Due to better endoplasmic reticulum proteost axis and decreased SOD1 aggregation, SIL1 overexpression provides considerable neuroprotection, whereas SIL1 deficiency worsens ALS pathogenesis	(Kikuchi et al. 2006; Bosco et al. 2010; Rozas et al. 2017)
11	Renal Cancer Carcinoma	Cell survival, drug resistance	It has been scientifically proven that GRP78 is significantly upregulated in Renal Cancer Carcinoma (RCC), promoting cell survival and inhibiting apoptosis. GRP78 overexpression induced sunitinib resistance of RCC cells by triggering the unfolded protein response	(Huang et al. 2019; Kumar et al. 2021; Shen et al. 2019)
12	Primary Sjögren's syndrome (pSS)	Autoimmunity, Inflammatory	Elevated activation of both β-arrestin2 and the GRP78-ATF6-CHOP apoptotic signaling pathway has been observed in patients with primary Sjögren's syndrome (pSS). This suggests that an hyperactivation of the GRP78-ATF6-CHOP pathway plays a role in the development of pSS, and β-arrestin2 promotes apoptosis in epithelial cells triggered by inflammation by acting through the GRP78-ATF6-CHOP pathway	(Huang et al. 2021a, b)
13	Renal tubulointerstitial fibrosis	Promotes fibrosis and inflammation	Jin reported that S100A16 expression is significantly increased in the cytoplasm during renal injury, making path for GRP78 entrance into the cytoplasm. The co-interaction of S100A16 with GRP78 promote the release of IRE1 in the cytoplasm	(Jin et al. 2021)
14	Retina degradation	Involved in the degradation of retinal cells	Studies have implicated GRP78 in the deterioration of the retina. Elevated levels of GRP78 is associated with the activation of glial cells and their neuroprotective function through the modulation of the unfolded protein response (UPR) during stressful conditions	(Park et al. 2021)
15	Pathogenesis of osteoarthritis (OA)	Contributes to the development and progression of OA	It has been suggested that GRP78 plays a role in the excessive growth of synovial cells in osteoarthritis (OA). Studies show that the presence of IL-1β, a pro-inflammatory cytokine, leads to an upregulation of GRP78, NF-κB (phosphorylation of the p65 subunit), IL-6, and PGE2 in primary synoviocytes. This increase in GRP78 expression is accompanied by enhanced polarization of macrophages towards the M1 phenotype. Moreover, when GRP78 is specifically knocked down, there is a significant reversal in the expression of downstream molecules associated with GRP78 and macrophage polarization induced by IL-1β	(Lee et al. 2021)
16	COVID-19 patients	Plays a role in the pathogenesis and severity of COVID-19	GRP78 is a crucial chaperone within the endoplasmic reticulum (ER) and its regulation is influenced by ATF4, either through direct or indirect mechanisms. In times of cellular stress, GRP78 becomes overexpressed and can be found on the surface of nearly all cells, where it serves as a receptor for the SARS-CoV-2 virus. A different clinical study, involving a significant number of participants, demonstrated that COVID-19 patients exhibited GRP78 levels approximately five times greater than those observed in the healthy control group Recent research has demonstrated that in addition to the receptor ACE2, csGRP78 can function as a co-receptor for the SARS-CoV-2 spike protein. In order to enter target cells more easily, csGRP78 forms a protein complex on the cell surface with the host cell receptor ACE2 and directly interacts with the SARSCoV-2 spike protein	(Shahriari-Felordi et al. 2022; Sabirli et al. 2021; Shin et al. 2021, 2022a; Carlos et al. 2021; Shin et al. 2022a, b)
17	Ovarian Cancer	Cell survival, angiogenesis, chemoresistance	GRP78 enhances cell survival, promotes angiogenesis, and contributes to resistance to chemotherapy in ovarian cancer. GRP78 enhances cell survival by activating pro-survival signaling pathways, such as the PI3K/AKT pathway. It also interacts with pro-apoptotic proteins to inhibit death	(Wu et al. 2021; Liu et al. 2013)
18	Myeloid leukemia (AML)	Implicated in the pathogenesis and progression of AML	In a recent study, a T cell that produces a chimeric antigen receptor (CAR) targeting GRP78, was developed. This approach demonstrated several advantages, including minimal T cell self-destruction after activation and genetic modification, as well as T cell differentiation dependent on the presence of the target antigen. The GRP78-CAR T cells effectively identified and eliminated AML (acute myeloid leukemia) cells that expressed GRP78, without harming healthy hematopoietic progenitor cells (HPCs). In vivo experiments also revealed significant effectiveness of GRP78-CAR T cells in combating AML. Furthermore, the study revealed high levels of GRP78 expression on the cell surface of AML patients' peripheral blood cells, and its overexpression in chronic lymphocytic leukemia patients' cells compared to normal B cells. Surface-expressed GRP78 was discovered to boost the generation of diverse cytokines with anti-tumor properties, including interferon (IFN)-γ, interleukin (IL)-2, tumor necrosis factor α (TNFα), and granulocyte–macrophage colony-stimulating factor (GM-CSF). Moreover, it exhibited a moderate elevation in the levels of interleukins -4, -5, -6, -10, and -13	(Hebbar et al. 2022)
19	Astrocytoma and Glioblastomas	Involved in tumor growth and progression	In astrocytoma’s and glioblastomas, the protein that is activated by GRP78 is called Akt (also known as protein kinase B). Akt is a key player in cell survival and growth pathways. The mechanism by which GRP78 activates Akt involves a process called phosphorylation. When GRP78 is overexpressed in cancer cells, it can bind to and activate a protein called PI3K (phosphoinositide 3-kinase). Activated PI3K then phosphorylates Akt, leading to its activation. The activation of Akt through GRP78 leads to an increase in the phosphorylation of procaspase-9. This phosphorylation event subsequently leads to a decrease in the levels of cleaved caspase 7, a protein involved in apoptosis. On the other hand, GRP78 can also interact with the proapoptotic protein BIK, as well as caspases 7 and 12, forming a complex. This complex formation serves to inhibit the release of cytochrome c from mitochondria and effectively limits the process of apoptosis	(Zhang et al. 2011)
20	ZIKV virus	Impacts ZIKV virus replication and pathogenesis	ZIKV viral domain III, which is involved in receptor binding, is capable of interacting with GRP78. Therefore, ZIKV binding, internalization, and replication in cells can be mediated by GRP78 to fulfill its function. Once ZIKV binds to GRP78, it triggers a series of events that facilitate viral entry into the cell. The virus is internalized through a process called endocytosis, where it is engulfed by the cell membrane and enclosed in a vesicle. This allows ZIKV to enter the cell's interior while remaining protected from the immune system. Once inside the cell, ZIKV can release its genetic material and start replicating. GRP78 may also play a role in facilitating viral replication by creating a favorable environment for viral protein synthesis and assembly	(Khongwichit et al. 2021; Elfiky and Ibrahim 2021; Turpin et al. 2020; Royle et al. 2020; Mufrrih et al. 2021)
21	Hepatitis B virus infectivity and antigen secretion	Regulates HBV infectivity and antigen secretion	In human hepatocellular cell line, the presence of GRP78 was confirmed in human hepatocellular cell line; interestingly, it is associated with preS2, a specific component of the hepatitis B virus (HBV). The study revealed that preS2 and GRP78 co-localize, indicating their proximity within the cells. Notably, preS2 selectively binds to the ATPase domain of GRP78, establishing a specific interaction between the two. This interaction has significant implications for both HBV infectivity and the secretion of HBV antigens. Moreover, the study demonstrated that GRP78's interaction with preS2 plays a role in promoting HBV replication. It achieves this by suppressing the expression of CHOP, a protein involved in the cellular stress response. By inhibiting CHOP, GRP78 enhances the survival of hepatocytes during persistent HBV infection. This finding shed light on the mechanism by which GRP78 influences HBV replication and suggests its importance in facilitating HBV-related cellular processes	(Suwanmanee et al. 2021)
22	Tongue cancer	Contributes to tumor growth and metastasis	In a study conducted by Lin et al., it was observed that the overexpression of GRP78/BiP is a strong indicator of a worse outcome in both precancerous and cancerous lesions, indicating a significant association with increased malignant potential in oral lesions. Another study by Kaira et al. analyzed 85 tongue cancer patients and found that elevated expression of GRP78/BiP independently predicts a poor prognosis in these patients. Moreover, GRP78/BiP exhibited significant associations with PERK expression, vascular invasion, glucose metabolism, and cell proliferation. Importantly, the expression of GRP78/BiP was markedly elevated in metastatic sites when compared to the primary sites	(Lin et al. 2010; Kaira et al. 2016)
23	Lung Cancer	Cell proliferation, inhibition of apoptosis	GRP78 in lung cancer enhances cellular proliferation, inhibits apoptosis, and imparts resistance to chemotherapy. It achieves these effects through various mechanisms. GRP78 stimulates cell proliferation by activating signaling pathways responsible for cell growth and survival, such as PI3K/AKT and MAPK/ERK pathways. It also suppresses apoptosis by interacting with proteins that promote cell death and inhibiting their function. Moreover, GRP78 facilitates the metastasis of lung cancer cells by promoting their invasiveness, migration, and invasion into surrounding tissues. This is accomplished through its interaction with proteins involved in epithelial-mesenchymal transition (EMT), a process enabling cancer cells to acquire characteristics associated with invasiveness and metastasis	(Du et al. 2019; Xia et al. 2021; Ning et al. 2022a, b; Tsai et al. 2007)
24	Pancreatic Cancer	ER stress, cell survival	Pancreatic cancer cells often face harsh microenvironments with limited nutrient and oxygen supply, leading to ER stress. GRP78 helps pancreatic cancer cells adapt to these conditions by activating the UPR and promoting cell survival. It also contributes to chemoresistance by regulating drug efflux transporters and modulating the activity of apoptosis-related proteins	(Lu et al. 2020; Tran et al. 2022)
25	Obesity & type 2 diabetes patients	Implicated in insulin resistance and metabolic dysfunction	GRP78 plays diverse roles in the development of obesity and type 2 diabetes by influencing ER stress, insulin signaling, inflammation, and lipid metabolism. It interacts with inflammatory signaling pathways, such as NF-κB and JNK, regulating the production of pro-inflammatory cytokines like TNF-α and IL-6, which contribute to chronic inflammation and insulin resistance. Additionally, GRP78 modulates lipid metabolism by interacting with transcription factors like SREBP-1c and PPARγ, affecting processes such as lipogenesis, adipocyte differentiation, and fatty acid handling. These mechanisms collectively contribute to insulin resistance and β-cell dysfunction observed in obesity and type 2 diabetes	(Pan et al. 2022; Han et al. 2015; Luo et al. 2022; Nourbakhsh et al. 2022)
26	Sperm Function	Essential for sperm maturation and fertilization	Inhibition of GRP78 has detrimental effects on the signaling of the PI3K/PDK1/AKT pathway in spermatozoa during capacitation. Additionally, suppressing GRP78 leads to adverse effects on sperm motility, kinematic parameters, capacitation state, cell viability, and abnormal tyrosine phosphorylation. These findings collectively suggest that the disruption of PI3K/PDK1/AKT signaling and tyrosine phosphorylation resulting from GRP78 suppression can have a negative impact on sperm function	(Lee et al. 2022b)
27	Osteosarcoma	Promote tumor growth, invasion and drug resistance	Osteosarcoma cells have elevated GRP78 expression andAKT activity is activated due to this. P-glycoprotein (P-gp), an important contributor to acquired multidrug resistance (MDR) in osteosarcoma, is consequentially produced in high levels as a result of AKT activation	(Zhang et al. 2022a, b)
28	Squamous cell carcinoma (SCC)	Contributes to tumor growth, invasion and metastasis	Overexpression of GRP78 in squamous cell carcinoma (SCC) contributes to its aggressive nature and unfavorable prognosis. It promotes cell survival, activates pro-survival pathways, induces chronic ER stress, stimulates angiogenesis, and enhances tumor invasion and metastasis. These mechanisms collectively drive the aggressive behavior of SCC. Targeting GRP78 and its associated pathways holds promise as a potential therapeutic strategy for managing this type of skin cancer. A recent study demonstrated that TMTC3 disrupts the interaction between PERK and GRP78, resulting in the activation of the PERK pathway. This activation facilitates the translocation of ATF4 to the nucleus, leading to increased transcriptional activity of ILEI. These findings suggest that TMTC3 promotes the GRP78/PERK signaling pathway during ER stress-induced epithelial-mesenchymal transition (EMT) in SCC. Targeting this pathway involving TMTC3 could offer new therapeutic opportunities for treating SCC	(Yuan et al. 2022)
29	Liver Cancer	Unfolded protein response, cell survival	GRP78 is implicated in liver cancer. It aids cell survival by activating pro-survival pathways, such as the PI3K/AKT and MAPK/ERK pathways. It also regulates the UPR to maintain protein homeostasis and cell survival. Additionally, GRP78 is associated with chemoresistance in liver cancer by modulating drug efflux pumps and anti-apoptotic pathways	(Su et al. 2010)
30	Melanoma	Unfolded protein response, cell survival	GRP78 promotes cell survival by activating pro-survival pathways and inhibiting apoptosis in melanoma cells. It interacts with and stabilizes anti-apoptotic proteins, such as Bcl-2 family members, which prevents the activation of apoptotic pathways. This leads to increased survival of melanoma cells	(Martin et al. 2010; Papalas et al. 2010; Lizardo et al. 2016; Sykes et al. 2016)
31	Brain Tumors	Tumor angiogenesis, cell survival, tumor invasion	GRP78 promotes tumor angiogenesis in brain tumors by regulating the expression of angiogenic factors. It also stimulates the production and secretion of vascular endothelial growth factor, a potent inducer of blood vessel formation. Increased GRP78 expression leads to elevated VEGF levels, which promote the growth of new blood vessels, providing nutrients and oxygen to tumor cells. Summarily, GRP78 aids tumor angiogenesis, promotes cell survival, and regulates invasion of brain tumors	(Ning et al. 2013; Sykes et al. 2016)
32	Bladder Cancer	Cell survival, chemotherapy resistance	GRP78 promotes tumor growth and proliferation in bladder cancer through multiple mechanisms. It interacts with cell surface receptors, such as integrins, promoting cell adhesion, migration, and invasion. GRP78 also activates key signaling pathways, including the PI3K/AKT and MAPK/ERK pathways, which enhance cell survival, proliferation, and angiogenesis. GRP78 is involved in the induction of EMT, a process where cancer cells acquire a mesenchymal phenotype with increased invasive properties. It regulates the expression of EMT-related markers, such as E-cadherin, N-cadherin, and vimentin, leading to enhanced migration and invasion of bladder cancer cells	(Udagawa et al. 2008)
33	Thyroid Cancer	Cell proliferation, inhibition of apoptosis	GRP78 activates pro-survival signaling pathways, such as the PI3K/AKT and MAPK/ERK pathways, in thyroid cancer cells. These pathways promote cell survival, proliferation, and resistance to apoptosis, leading to enhanced tumor growth and survival. It also influences the tumor microenvironment in thyroid cancer by promoting angiogenesis and inflammation. It interacts with vascular endothelial growth factor (VEGF) and matrix metalloproteinases (MMPs), contributing to the formation of new blood vessels and facilitating tumor growth and metastasis	(Luo et al. 2018; Mahadevan et al. 2011; Zhao et al. 2020)
34	Kidney Cancer	Unfolded protein response, cell survival	GRP78 plays a role in maintaining protein homeostasis by activating the UPR, which helps kidney cancer cells adapt to cellular stress. It promotes cell survival and supports tumor progression by inhibiting apoptosis and promoting cell proliferation in the presence of UPR activation	(Abhishek et al. 2017; Shi et al. 2019; Trink et al. 2023)
35	Esophageal Cancer	Cell survival, chemotherapy resistance	GRP78 is active in modulating cell migration, invasion, and metastasis in esophageal cancer. It influences the expression and activity of matrix metalloproteinases (MMPs), enzymes involved in extracellular matrix remodeling and tumor cell invasion. Additionally, GRP78 can interact with cell adhesion molecules and components of the cytoskeleton, affecting cell adhesion, migration, and invasion processes	(Zhang et al. 2020)
36	Skin Disease	Regulation of osteoblast and osteoclast	GRP78 regulates the activation of immune cells, such as macrophages and dendritic cells, leading to the production of pro-inflammatory cytokines. GRP78 contributes to the proliferation and migration of skin cells during wound healing. However, GRP78 can heighten cell survival and proliferation, which can result into development and growth of skin cancer. Pro-survival signaling pathways, such as the PI3K/AKT and MAPK/ERK pathways, that enhance cell survival and stimulate cell division is also activated by GRP78	(Park et al. 2019)
37	Eye Diseases	Participation in retinal degeneration	GRP78 plays a role in modulating endoplasmic reticulum (ER) stress and apoptotic pathways in retinal cells. It helps maintain ER homeostasis and prevents the accumulation of misfolded proteins, which can trigger cellular stress and apoptosis in the retina. In eye diseases such as diabetic retinopathy and retinal ischemia, dysregulation of apoptosis can contribute to retinal cell damage and vision loss. GRP78 interacts with pro-apoptotic factors and inhibits apoptotic signaling, promoting cell survival	(Kroeger et al. 2019)
38	Reproductive Disorders	Regulation of fertility, hormone secretion, and development in the reproductive system	GRP78 is involved in the regulation of reproductive processes, including follicular development, ovulation, and hormone secretion. It plays a role in maintaining proper ER function and protein folding in the reproductive tissues. Disruption of GRP78 function can lead to reproductive system dysfunction, affecting fertility and hormone balance	(Zhang 2017; Hebert-Schuster et al. 2018; Lee et al. 2022c)
39	Blood Disorders	Involvement in hematological malignancies, particularly promoting leukemic cell survival and drug resistance	GRP78 contributes to the survival and proliferation of leukemic cells by regulating ER stress response, promoting anti-apoptotic pathways, and modulating drug resistance mechanisms. Its overexpression can confer a survival advantage to leukemic cells and limit the effectiveness of anti-cancer treatments	(Mozos et al. 2011; Best et al. 2019)
40	Autoinflammatory	Modulation of inflammatory responses and cytokine production in autoinflammatory disorders	GRP78 is involved in the regulation of immune signaling pathways and inflammatory responses. It can modulate the production of pro-inflammatory cytokines and influence the activation of immune cells. Dysregulation of GRP78 expression or function may contribute to excessive inflammation and the pathogenesis of autoinflammatory disorders	(Van Kempen et al. 2015; Agyemang et al. 2015)
41	Neuroblastoma	Cell survival, chemoresistance	GRP78 exhibits anti-apoptotic properties by suppressing apoptosis, or programmed cell death. It interacts with and regulates the activity of several pro-apoptotic proteins, such as caspases, BAX, and PERK (protein kinase R-like endoplasmic reticulum kinase). Through these interactions, GRP78 prevents the activation of apoptotic pathways, thereby promoting cell survival and contributing to neuroblastoma progression	(Zhang et al. 2016a, b; Hsu et al. 2005)
42	Wilms Tumor	Cell survival, chemotherapy resistance	Facilitates cell survival and confers resistance to chemotherapy. GRP78 has anti-apoptotic properties by preventing apoptosis, a programmed cell death process. It interacts with and modulates the activity of pro-apoptotic proteins such as caspases, BAX, and PERK (protein kinase R-like endoplasmic reticulum kinase). By inhibiting the activation of apoptotic pathways, GRP78 can promote cell survival and potentially contribute to Wilms tumor progression	(Wang et al. 2017a, b)
43	Cardiovascular disease	Endothelial dysfunction, oxidative stress, inflammation, apoptosis	Cardiovascular disease refers to a group of disorders affecting the heart and blood vessels, including conditions like coronary artery disease, heart failure, and stroke. It is a leading cause of death worldwide. Cell surface GRP78 is involved in multiple mechanisms contributing to cardiovascular disease. It can promote inflammation, angiogenesis, and platelet aggregation. It can also interact with various receptors, such as toll-like receptors (TLRs), integrins, and vascular endothelial growth factor receptor 2 (VEGFR2), leading to downstream signaling pathways that influence cell survival, proliferation, and migration In the context of myocardial infarction (MI), commonly known as a heart attack, cell surface GRP78 has also emerged as a relevant factor. Increased expression of GRP78 on the cell surface has been observed in ischemic myocardium. In MI, cell surface GRP78 contributes to cardiac injury through various mechanisms. It promotes myocardial apoptosis, inflammation, and fibrosis, thereby exacerbating tissue damage and impairing cardiac function. Moreover, GRP78 interacts with integrins and activates downstream signaling pathways, leading to increased fibrotic tissue formation. These combined effects of cell surface GRP78 contribute to the adverse remodeling processes seen in MI	(Wang et al. 2018; Bhattacharjee et al. 2005; Sato et al. 2010b; Girona et al. 2019; Liu et al. 2003; Lee 2007; Sha and Jiang 2023; Hardy and Raiter 2010)

### Novel approaches employed in downregulating GRP78 implicated in various diseases

Several researchers have been actively exploring the possibilities of targeting GRP78 downregulation in cancer therapeutic strategy. These efforts aim to restore normal cellular function and counteract the detrimental effects associated with GRP78 dysregulation. Recent publications have shed light on various innovative approaches employed in targeting GRP78 expression or function. Downregulation of GRP78 expression has been investigated as a potential therapeutic strategy in several diseases(Luo et al. 2018; Wang et al. 2017a, b). In prostate cancer, Lu et al. utilized RNA interference technology to downregulate GRP78 and GRP74 expression in the PC-3 cell line, and this result in reduced cell migration and induction of apoptosis(Lu et al. 2019). Another study focused on inhibiting the VEGF/GRP78 axis using the sFLT01 protein in human prostate cancer cells (DU145). The researchers observed downregulation of GRP78 and matrix metallopeptidase proteins 2&9 (MMP2&9) transcript levels, along with increased expression of tissue inhibitor of metalloproteinase proteins 1&2 (TIMP1&2), suggesting that sFLT01 inhibited cancer cell proliferation and invasiveness (Taghizadeh et al. 2021).

In breast cancer, a proteomic approach identified cell surface GRP78 and Dermcidin (DCD) as cooperative regulators of breast cancer cell migration through Wnt signaling. The interaction between GRP78 and DCD was found to be involved in the regulation of stem cell and cancer cell migration(Lager et al. 2021). Additionally, upregulating ATAD3A in colorectal cancer cells was shown to stabilize GRP78, reduce endoplasmic reticulum (ER) stress, and decrease chemotherapy-induced cancer cell death(Huang et al. 2021a, b). Cardiac glycosides (CGs), such as Lanatoside C (LanC), have been investigated for their inhibitory effect on GRP78 activation induced by ER stress in pancreatic cancer cells. The inhibition of GRP78 activation was associated with apoptosis induction in pancreatic cancer cells(Ha et al. 2021). Furthermore, Suppressor of cytokine signaling-3 (SOCS3) was found to target GRP78 ubiquitination for proteasomal degradation, thereby blocking ER stress and mitophagy pathways in cardiac hypertrophy, which could potentially prevent heart failure(Liu et al. 2021).

**Fig. 6:**
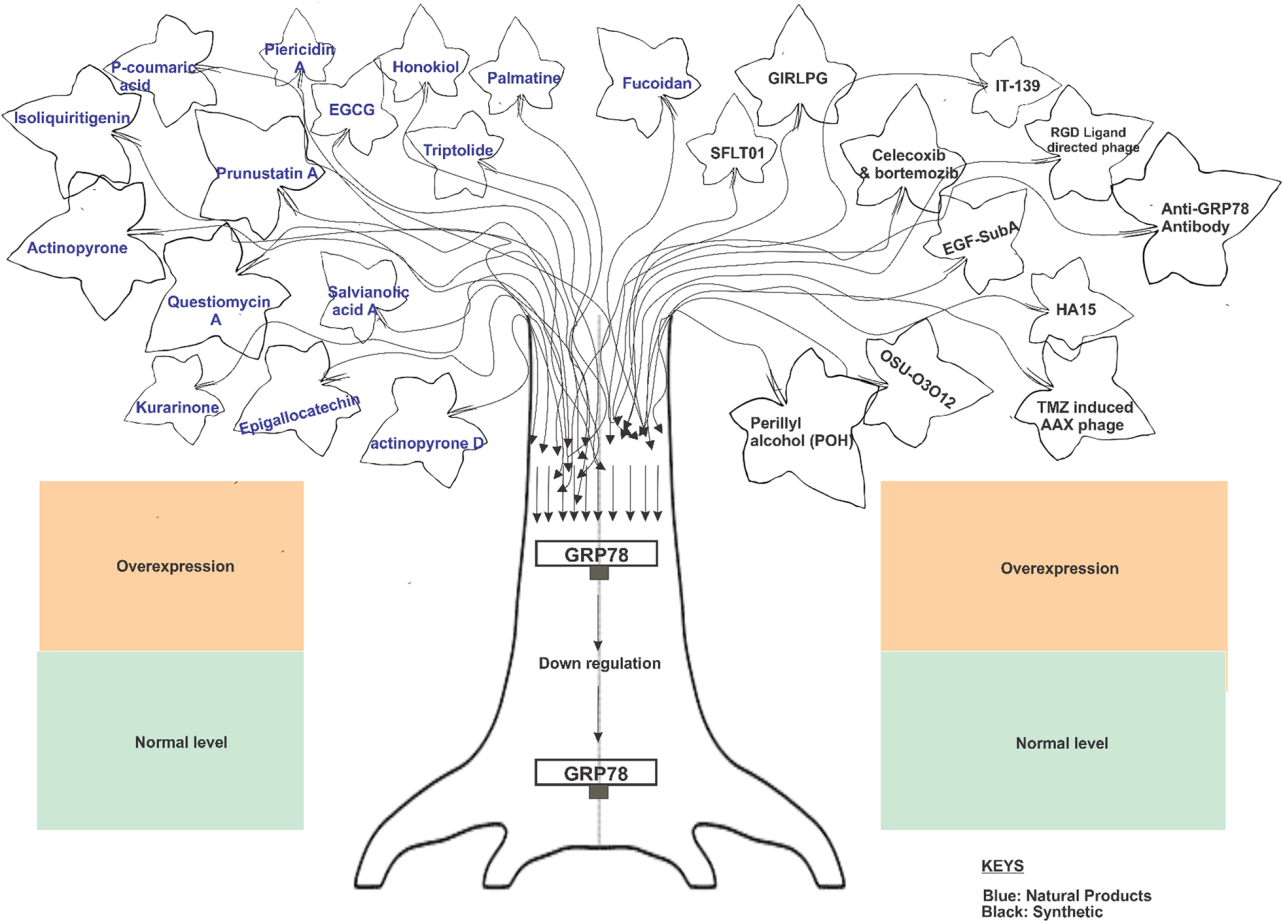
Overview of Synthetic and Natural Products that has been employed in Downregulating GRP78 in various cancers. Natural and synthetic compounds with anticancer properties that suppress GRP78 induction has been reported below; however, they exert pleiotropic effects

Traditional Chinese Medicine (TCM) treatments have also been explored for their ability to downregulate GRP78 expression. TCMSini's San (SNS), used for depression-related symptoms, was shown to attenuate GRP78 expression, preventing its interaction with LRP5 on the cell surface and inhibiting breast cancer stem cell signaling through the Wnt and β-catenin pathway(Zheng et al. 2021; Liu et al. 2022; Sadeghipour et al. 2022). Another TCM remedy, Ai Du Qing (ADQ), downregulated GRP78 expression, leading to the degradation of β-catenin and attenuating chemoresistance in breast cancer cells (Liao et al. 2021). Palmatine (Fig. 6), a plant-derived compound with various protective properties, was found to downregulate GRP78 expression in a streptozotocin-induced diabetic rat model. Palmatine treatment inhibited the upregulation of GRP78 in the pancreas, suggesting its potential as a therapeutic agent for diabetes(Okechukwu et al. 2021). In the field of immunotherapy, chimeric antigen receptor (CAR) T cells targeting cell surface GRP78 (GRP78.1x, GRP78.2x, and GRP78.3 × CAR T cells) demonstrated potent anti-tumor activity against acute myeloid leukemia (AML) cells. The CAR T cells induced the production of anti-tumor cytokines and effectively suppressed tumor progression in preclinical models (Yu et al. 2022) (Fig. 6).Recently, the activation of GRP78 ATPase by ZBM-H has been shown to suppress A549 lung cancer cell migration by promoting the degradation of integrin β4 (ITGB4). ZBM-H treatment resulted in decreased ITGB4 protein levels and inhibited the epithelial-mesenchymal transition (EMT) process in A549 cells. The activation of GRP78 ATPase by ZBM-H also facilitated the interaction between annexin A7 (ANXA7) and heat shock cognate 70 kDa protein (Hsc70), which contributed to the regulation of selective autophagy and degradation of ITGB4(Ning et al. 2022a, b).

These studies highlight various novel approaches employed to downregulate GRP78 expression and investigate its implications in different diseases. The use of RNA interference technology, protein inhibitors, traditional Chinese medicine, cardiac glycosides, and immunotherapy strategies targeting cell surface GRP78 have shown promising results in inhibiting tumor cell proliferation, migration, and inducing apoptosis. Additionally, the modulation of GRP78 expression has been explored in the context of diabetes, cardiac hypertrophy, and psychological stress-related diseases. Targeting GRP78 and exploring its regulatory mechanisms offer potential avenues for therapeutic interventions in cancer, diabetes, cardiovascular diseases, and other conditions associated with GRP78 dysregulation. Further research is needed to elucidate the underlying mechanisms and optimize the therapeutic strategies targeting GRP78 to improve patient outcomes.

### Agents that target cell surface GRP78

Agents that target cell surface GRP78 include specific peptides, both conjugated and unconjugated, as well as the human plasminogen factor kringle 5. These agents have demonstrated efficient targeting of cell surface GRP78. Additionally, antibodies against cell surface GRP78 have been developed with diverse mechanisms to induce cancer cell death and suppress GRP78-mediated oncogenic signaling(Lee 2014; Hernandez and Cohen 2022). Numerous strategies have been employed to reduce cell surface GRP78 levels, and various inhibitory agents have been investigated. The table below provides a brief overview of phytochemicals, peptides, and antibodies that have shown inhibitory effects against cell surface GRP78 (Table 3) (Elfiky et al. 2020; Shimizu et al. 2022).

**Table 3:** Shows various Agents that target cell surface GRP78

Agents that target cell surface GRP78	Types	References
Phytochemicals	Fungi: Fungi, such as mushrooms, contain p-Coumaric acid and caffeic acid, both of which have been shown to have a binding affinity for the GRP78 SBD beta region in computational models. These compounds have been proposed as potential inhibitors for GRP78 to counteract its overexpression in cancer cells Epigallocatechin3gallate: Another compound of interest is epigallocatechin-3-gallate (EGCG), a polyphenol found in green tea. EGCG has demonstrated an anti-proliferative effect on melanoma and breast cancer cells. Moreover, EGCG has been found to directly interact with the ATP-binding site of GRP78, effectively inhibiting GRP78 function by competing with ATP binding. This interaction has the potential to disrupt the proper folding and processing of proteins, leading to cellular stress and potential cancer cell death	(Elfiky 2021; Nihal et al. 2005; Ermakova et al. 2006)
Peptides	Several peptides and peptide conjugates have been investigated for their ability to interact with GRP78 and exert anti-cancer effects GMP1 peptide: The GMBP1 peptide has been utilized to reverse multidrug resistance (MDR) in stomach cancer. It functions by utilizing GRP78 as a receptor, facilitating its internalization into cancer cells through the transferrin-related pathway. By targeting GRP78, GMBP1 disrupts the MDR mechanism, potentially sensitizing cancer cells to chemotherapy drugs Gonadotropin-releasing hormone analogs (GnRHa): Gonadotropin-releasing hormone analogs (GnRHa) have also been shown to block GRP78 and prevent the proliferation of defective cells. This disruption of GRP78 function triggers apoptosis, or programmed cell death, in these cells. GnRHa holds promise as a therapeutic agent for cancers characterized by GRP78 dysregulation Pep42-taxol and Pep42-doxorubicin: In the case of highly metastatic human melanoma cells, pep42-taxol and pep42-doxorubicin conjugates have been developed. These conjugates specifically bind to GRP78, resulting in the death of GRP78-expressing cancer cells in vitro. By selectively targeting GRP78, these conjugates offer a potential strategy to combat metastatic melanoma WIFPWIQL peptide: Another peptide of interest is the WIFPWIQL peptide. This peptide binds to GRP78 on cancer cells, and when fused with SubA, it forms an effective anticancer drug. The WIFPWIQL peptide is responsible for recognizing and internalizing GRP78 into cancer cells, while SubA, a bacterial cytotoxin, exerts a toxic effect by cleaving GRP78 inside the cell, inducing apoptosis	(Wang et al. 2015; Weng et al. 2014; Ibrahim et al. 2019; Beddoe et al. 2010; Zhang et al. 2016a, b)
Monoclonal Antibodies	α 2-macroglobulin: The interaction between activated α2-macroglobulin and GRP78 on the surface of human prostate cancer cells plays a significant role in stimulating cell proliferation. This binding activates signaling cascades within the cells. Specifically, the N-terminal portion of cell surface GRP78 is connected to α2-macroglobulin, triggering the activation of the Akt pathway. This activation, in turn, blocks apoptotic pathways and promotes cell growth. This mechanism highlights the involvement of GRP78 in prostate cancer cell proliferation Mouse Monoclonal Antibody C107 & C38: Mouse monoclonal antibodies C107 and C38 recognize the C-terminal region of murine GRP78, which is exposed on the cell membrane. Upon binding, these antibodies exert their effects on melanoma cells. Specifically, they suppress the Akt/PI3K proliferative pathway. By targeting the exposed C-terminal region of GRP78, these antibodies interfere with the signaling cascades that promote cell growth and proliferation, offering potential therapeutic applications in melanoma treatment The binding of activated α2-macroglobulin to GRP78 and the interaction between mouse monoclonal antibodies and GRP78 highlight the intricate involvement of GRP78 in signaling pathways and its potential as a therapeutic target. Further research is needed to explore the precise mechanisms and clinical applications of these interactions	(Misra et al. 2009; de Ridder et al. 2012)
Anti-GRP78 autoantibodies	Anti-GRP78 autoantibodies have emerged as potential therapeutic agents for targeting cell surface GRP78 in various diseases, including cancer. The groundbreaking work by Wadih Arap and Renata Pasqualini, initially conducted at the University of Texas MD Anderson Cancer Center, played a pivotal role in identifying these autoantibodies in patients with cancer. In their original study published in the Proceedings of the National Academy of Sciences in 1998, titled "Phage display selection of ligand peptides targeting atherosclerotic lesions," Arap and Pasqualini employed phage display technology to identify peptides that specifically bound to atherosclerotic lesions in mice. Although this study primarily focused on atherosclerosis, it set the stage for subsequent investigations into the role of cell surface GRP78 and anti-GRP78 autoantibodies in cancer. Subsequently, Arap and Pasqualini continued their research at Rutgers University, delving deeper into the significance of anti-GRP78 autoantibodies in cancer. They discovered that certain autoantibodies targeted the N-terminal region of cell surface GRP78 and exhibited an unexpected effect by accelerating tumor growth. These autoantibodies appeared to enhance tumor cell survival and invasiveness by activating signaling pathways that promoted angiogenesis and immune evasion. Simultaneously, Arap and Pasqualini's research also revealed the presence of C-terminal anti-GRP78 autoantibodies in cancer patients. Unlike the N-terminal autoantibodies, these C-terminal autoantibodies exhibited tumor growth inhibitory effects. They were shown to block the growth of tumor cells and induce cell death, suggesting their potential as therapeutic agents for targeting cell surface GRP78 in cancer treatment. The identification of anti-GRP78 autoantibodies by Arap and Pasqualini provided a valuable insight into the complex role of cell surface GRP78 in cancer progression. These autoantibodies, particularly the C-terminal variants, demonstrated the therapeutic potential of targeting cell surface GRP78. By blocking the growth-promoting activities of GRP78 and inducing tumor cell death, they offered a novel strategy for combating cancer. The pioneering studies conducted by Arap and Pasqualini not only identified anti-GRP78 autoantibodies in cancer patients but also provided a foundation for subsequent investigations into the functional implications of these autoantibodies. Their work sparked further research by numerous scientists and research groups to explore the therapeutic potential of targeting cell surface GRP78 in cancer and other diseases Recent studies published in JCI Insight by Crane et al. highlighted the role of anti-GRP78 autoantibodies in promoting atherosclerosis. These autoantibodies were found to bind to cell surface GRP78 on endothelial cells within atherosclerotic lesions, leading to the activation of inflammatory and pro-atherogenic signaling pathways. The research demonstrated that targeting cell surface GRP78 or interfering with the binding of autoantibodies to GRP78 could attenuate atherosclerotic lesion formation, suggesting potential therapeutic avenues for treating cardiovascular diseases The seminal studies by Arap and Pasqualini, as well as subsequent investigations by the Pizzo and Gonzalez-Gronow group at Duke and the Al-Hashimi group at McMaster, have deepened our understanding of the functional significance of cell surface GRP78 and its potential as a therapeutic target in various diseases. Ongoing research continues to uncover the complex mechanisms underlying cell surface GRP78's roles in disease progression and explore its therapeutic implications	(Crane et al. 2018; Lebeau et al. 2021; Al-Hashimi et al. 2017; Mintz et al. 2003; Arap et al. 2004; Sato et al. 2010a; D’Angelo et al. 2018)

**Fig. 7:**
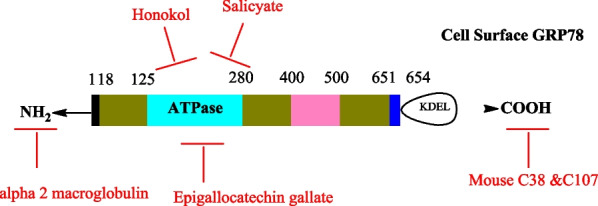
Depicts some significant agents that specifically target GRP78 on cell surface

Despite, various strategies employed, there are some compounds that are site specific when binding. Several compounds like Honokiol, Salicylate and epigallocatechin gallate; directly bind to ATP binding domain to inactivate GRP78 in cancer(Ermakova et al. 2006; Martin et al. 2013). More so, alpha 2 macroglobulin and Mouse C38 and C107 binds with cell surface GRP78 at the N and C terminal respectively (Misra et al. 2009)(Fig. 7 and Table 4).

**Table 4:** Shows site specific binding compounds

Site specific compound	Binding site	References
Honokiol	ATP binding domain	(Ermakova et al. 2006)
Salicylate	ATP binding domain	(Ermakova et al. 2006)
Epigallocatechin gallate (EGCG)	ATP binding domain	(Martin et al. 2013)
Alpha 2 macroglobulin	Cell surface GRP78 (N-terminal)	(Misra et al. 2009)
Mouse C38	Cell surface GRP78 (C-terminal)	(Misra et al. 2009)
Mouse C107	Cell surface GRP78 (C-terminal)	(Misra et al. 2009)

### Clinical significance of GRP78

The clinical significance of glucose-regulated protein 78 (GRP78) in cancer is profound, as it participates in several tumor biology-related processes, suggesting its potential as a therapeutic target. GRP78 holds significance in three key aspects: tumor therapy, enhancement of therapeutic benefits, and as a prognostic biomarker.

Firstly, GRP78 can serve as a target for tumor therapy. Its overexpression in cancer cells has been consistently observed and is associated with various aspects of tumor progression, including cell survival, resistance to apoptosis, angiogenesis promotion, and facilitation of epithelial-mesenchymal transition (EMT). Targeting GRP78 with selective inhibitors or antibodies offers the potential to disrupt its pro-survival functions, sensitize cancer cells to conventional therapies, and overcome treatment resistance. By inhibiting GRP78, it becomes possible to interfere with critical cellular processes essential for cancer cell survival and growth, thereby enhancing the effectiveness of cancer treatment. Secondly, the creation of unfolded protein response (UPR)-related proteins, particularly GRP78, can enhance the therapeutic benefits of drugs commonly used in clinical practice. The UPR is a cellular stress response mechanism activated in cancer cells under conditions of increased protein folding demand and nutrient deprivation. GRP78 is a key regulator of the UPR and assists in maintaining cellular homeostasis during stress. Modulating the UPR through GRP78 can improve the efficacy of anticancer drugs by increasing their cytotoxic effects on cancer cells. This approach has the potential to enhance treatment outcomes and overcome drug resistance. Lastly, GRP78 can serve as a biomarker for evaluating cancer patients and predicting prognosis. The expression levels of GRP78 in tumor tissues or body fluids can be indicative of disease progression and patient outcomes. Assessing GRP78 as a diagnostic or prognostic biomarker holds promise for early cancer detection and risk stratification. By evaluating GRP78 expression, it becomes possible to identify high-risk patients who may benefit from aggressive treatment strategies or personalized therapies. Additionally, monitoring changes in GRP78 levels during the course of treatment can provide valuable information on treatment response and disease status.

In preclinical studies, selective inhibitors or antibodies against GRP78 have shown promising results in disrupting its pro-survival functions and sensitizing cancer cells to conventional therapies (Lee 2007). For instance, a study by Chen et al. (2022) demonstrated that an anti-GRP78 antibody, PAT-SM6, effectively inhibited tumor growth in melanoma mouse models(Chen et al. 2022). Furthermore, combination therapies involving GRP78 targeting have been investigated to enhance treatment response. Combining GRP78 inhibition with conventional therapies or emerging treatment modalities, such as immunotherapy, has shown synergistic effects, leading to improved outcomes (Lev et al. 2017). These approaches hold great potential for overcoming treatment resistance and improving patient survival. One approach to improving therapeutic outcomes is by utilizing UPR-related proteins, including GRP78, to enhance drug delivery and sensitize cancer cells to treatment. For instance, Zhao et al. (2015) developed a nanoparticle-based drug delivery system that specifically targets GRP78-expressing cancer cells, resulting in improved drug efficacy and reduced systemic toxicity (Zhao et al. 2015). By exploiting the UPR pathway, such strategies have the potential to optimize the effectiveness of conventional chemotherapeutic agents and overcome drug resistance mechanisms.

Despite the challenges in comprehending the precise role of csGRP78 in cancer, the targeting of csGRP78 has shown promise in preclinical and early clinical studies. PAT-SM6, an anti-GRP78 antibody, has demonstrated potent anti-tumoral activity in preclinical models and has progressed to phase I clinical trials in patients with malignant melanoma (Hensel et al. 2013). Further investigations are warranted to explore the therapeutic potential of csGRP78-targeted strategies and to gain a deeper understanding of its functional implications in different cancer types.

## Conclusion and a Glimpse into the future

In conclusion, this study has provided a systematic breakdown and pictorial evidence showcasing the correlation between glucose-regulated protein 78 (GRP78) and various types of cancer. The consistent upregulation of GRP78 in cancer cells has been associated with tumor initiation, growth, metastasis, and resistance to therapy. The visual representation of GRP78 expression patterns in different cancer types strengthens its clinical relevance. GRP78 plays a crucial role in cancer development and progression by promoting cell survival, inhibiting apoptosis, facilitating angiogenesis, inducing epithelial-mesenchymal transition (EMT), and conferring therapy resistance. The extensive research supporting these observations highlights the potential of GRP78 as a therapeutic target in cancer treatment. The findings discussed here pave the way for several exciting future perspectives that could contribute to the development of effective cancer therapies. Firstly, there is a need to explore the therapeutic targeting of GRP78. The development of selective inhibitors or antibodies against GRP78 could disrupt its pro-survival functions and sensitize cancer cells to conventional therapies, potentially overcoming treatment resistance and improving patient outcomes. Secondly, investigating combination therapies involving GRP78 targeting is crucial. Assessing the synergistic effects of GRP78 inhibition with conventional therapies or emerging treatment modalities like immunotherapy may enhance treatment response and minimize the likelihood of relapse. Thirdly, exploring the utility of GRP78 as a diagnostic or prognostic biomarker holds promise. Further research is required to evaluate the clinical applicability of GRP78 in early cancer detection and risk stratification, enabling timely intervention and improved patient outcomes. Additionally, unraveling the intricate molecular mechanisms underlying GRP78's role in cancer progression is imperative. This knowledge will provide valuable insights into potential downstream targets and signaling pathways that can be exploited for therapeutic intervention. Finally, preclinical studies using in vitro and in vivo models should be conducted to validate the efficacy and safety of GRP78-targeted therapies before clinical translation. These studies will help optimize treatment strategies and assess potential side effects. In summary, the correlation between GRP78 and various cancers presents a promising avenue for future research and therapeutic interventions. By focusing on GRP78 as a target, we have the potential to overcome treatment resistance and improve outcomes for cancer patients. However, further investigation and validation are required to fully harness the therapeutic potential of GRP78 inhibition in clinical practice.

It is worth noting that GRP78 and other components of the unfolded protein response (UPR) also play important roles in autoimmune disorders and neurological disorders. Understanding the processes through which cell surface GRP78 functions will provide crucial insights into not only cancer but also these other disease areas. The overexpression of cell surface GRP78 in cancer makes it a potential target for tumor therapy(Farshbaf et al. 2020). Exploring new approaches that combine drugs promoting GRP78 translocation to the cell surface with peptides or small compounds targeting GRP78 could significantly enhance the efficacy of cancer treatments. Currently, two GRP78 antibodies, PAT-SM6 and Bold-100, have undergone testing in human clinical trials (Hensel et al. 2013; Rasche et al. 2015; Burris et al. 2016). Initial studies on PAT-SM6 in melanoma tumor-bearing mice showed potent anti-tumoral activity, and a phase I clinical trial was conducted in patients with malignant melanoma to assess the safety and anti-tumor effectiveness of the antibody (Hensel et al. 2013).

Furthermore, Bold-100 also known as IT-139, NKP1339) is a small ruthenium-based compound that specifically targets GRP78. By inhibiting GRP78, Bold-100 aims to disrupt the protective mechanisms of cancer cells, making them more vulnerable to standard cancer treatments such as chemotherapy and radiation therapy (Burris et al. 2016). The rationale behind targeting GRP78 is that it is overexpressed on the surface of cancer cells, providing them with enhanced survival advantages, including resistance to apoptosis (programmed cell death) and increased tolerance to stress conditions. By blocking GRP78, Bold-100 intends to sensitize cancer cells to treatment-induced cell death. Bold Therapeutics has conducted preclinical and early clinical studies to evaluate the safety and efficacy of Bold-100 in various types of cancers (Yoo et al. 2012).

Bold-100 is currently undergoing a global Phase 2 clinical trial for the treatment of advanced gastrointestinal cancers, including bile duct, colon, gastric, and pancreatic cancers. The trial, registered as NCT04421820, involves six sites in Canada, two in the United States, and five in South Korea. Promising results from the Phase 2 study in advanced colorectal cancer were presented at the American Association for Cancer Research (AACR) conference in April 2023. The data demonstrated a clinically significant improvement when compared to standard of care treatments. Specifically, among patients in the 3rd line and beyond with advanced colorectal cancer (n = 17), the study revealed a median Progression-Free Survival (PFS) of 4.7 months, Overall Survival (OS) of 9.8 months, and an Overall Response Rate (ORR) of 13%. The potential of Bold-100 has been recognized by the U.S. Food and Drug Administration (FDA), which has granted Orphan Drug Designations (ODDs) for gastric and pancreatic cancers. It is anticipated that in 2023, Bold-100 will also receive breakthrough therapy designations (BTDs) for colorectal cancer and potentially other indications. As demonstrated in this review, various solid tumor cell types express GRP78 on their surface, making them potential candidates for future clinical studies. Utilizing the GRP78 antibody in tumors where other antibody-based therapies are already available could offer clinical benefits through the development of mechanisms that enable targeted drug delivery.

Conclusively, further research and exploration are needed to fully exploit the therapeutic potential of GRP78 inhibition in cancer treatment, as well as to understand its implications in autoimmune and neurological disorders.

## Data Availability

All data generated or analyzed during this study are included in this published article. Resources: https://www.clinicaltrials.gov/ct2/show/NCT01727778**,** https://classic.clinicaltrials.gov/ct2/show/NCT04421820.

## Abbreviations

AD: Alzheimer's disease
AML: Acute myeloid leukemia
ATF6: Activating transcription factor 6
BiP: Binding immunoglobulin Protein
CAR: Chimeric antigen receptor
CsGRP78: Cell surface glucose regulatory protein
DCD: Dermcidin
EGCG: Epigallocatechin-3-gallate
EMT: Epithelial-mesenchymal transition
ER: Endoplasmic reticulum
GnRHa: Gonadotropin-releasing hormone analogs
GM-CSF: Granulocyte–macrophage colony-stimulating factor
GRP78: Glucose regulatory protein
GSK-3: Glycogen synthase kinase-3
HBV: Hepatitis B virus
HDAC1: Histone deacetylase 1
HPCs: Hematopoietic progenitor cells
HSP70: Heat shock protein 70
IRE1: Inositol requiring enzyme 1
JNK: JUN amino-terminal kinases
LanC: Lanatoside C
MDR: Multidrug resistance
MI: Myocardial infarction
MMPs: Matrix metalloproteinases
OA: Osteoarthritis
PERK: Protein kinase R-like endoplasmic reticulum kinase
P-gp: P-glycoprotein
PI3K: Phosphoinositide 3-kinase
pSS: Primary Sjögren's syndrome
SNc: Substantia nigra pars compacta
TCM: Traditional Chinese Medicine
TNFα: Tumor necrosis factor α
UPR: Unfolded protein response
